# Patient-Centered Chronic Wound Care Mobile Apps: Systematic Identification, Analysis, and Assessment

**DOI:** 10.2196/51592

**Published:** 2024-03-21

**Authors:** Tassilo Dege, Bernadette Glatzel, Vanessa Borst, Franziska Grän, Simon Goller, Caroline Glatzel, Matthias Goebeler, Astrid Schmieder

**Affiliations:** 1 Department of Dermatology, Venereology and Allergology, University Hospital Würzburg, Würzburg, Germany; 2 Department of Computer Science, University of Würzburg, Würzburg, Germany

**Keywords:** chronic wounds, chronic leg ulcers, mobile applications, evaluation, mental health, Mobile Application Rating Scale, System Usability Scale, affinity for technology interaction, ATI, teledermatology, disease management, health app, skin, eHealth, telemedicine, mHealth, mobile health, app, apps, applications, quality, rating, wound, wounds, chronic, ulcer, ulcers, sore, sores, dermatology, chronic wound

## Abstract

**Background:**

The prevalence of chronic wounds is predicted to increase within the aging populations in industrialized countries. Patients experience significant distress due to pain, wound secretions, and the resulting immobilization. As the number of wounds continues to rise, their adequate care becomes increasingly costly in terms of health care resources worldwide. eHealth support systems are being increasingly integrated into patient care. However, to date, no systematic analysis of such apps for chronic wounds has been published.

**Objective:**

The aims of this study were to systematically identify and subjectively assess publicly available German- or English-language mobile apps for patients with chronic wounds, with quality assessments performed by both patients and physicians.

**Methods:**

Two reviewers independently conducted a systematic search and assessment of German- or English-language mobile apps for patients with chronic wounds that were available in the Google Play Store and Apple App Store from April 2022 to May 2022. In total, 3 apps met the inclusion and exclusion criteria and were reviewed independently by 10 physicians using the German Mobile App Rating Scale (MARS) and the System Usability Scale (SUS). The app with the highest mean MARS score was subsequently reviewed by 11 patients with chronic wounds using the German user version of the MARS (uMARS) and the SUS. Additionally, Affinity for Technology Interaction (ATI) scale scores were collected from both patients and physicians.

**Results:**

This study assessed mobile apps for patients with chronic wounds that were selected from a pool of 118 identified apps. Of the 73 apps available in both app stores, 10 were patient oriented. After excluding apps with advertisements or costs, 3 apps were evaluated by 10 physicians. Mean MARS scores ranged from 2.64 (SD 0.65) to 3.88 (SD 0.65) out of 5, and mean SUS scores ranged from 50.75 (SD 27) to 80.5 (SD 17.7) out of 100. *WUND APP* received the highest mean MARS score (mean 3.88, SD 0.65 out of 5) among physicians. Hence, it was subsequently assessed by 11 patients and achieved a similar rating (uMARS score: mean 3.89, SD 0.4 out of 5). Technical affinity, as measured with the ATI scale, was slightly lower in patients (score: mean 3.62, SD 1.35 out of 6) compared to physicians (score: mean 3.88, SD 1.03 out 6).

**Conclusions:**

The quality ratings from physicians and patients were comparable and indicated mediocre app quality. Technical affinity, as assessed by using the ATI scale, was slightly lower for patients. Adequate apps for patients with chronic wounds remain limited, emphasizing the need for improved app development to meet patient needs. The ATI scale proved valuable for assessing technical affinity among different user groups.

## Introduction

### Background

Aging societies in industrialized nations are experiencing an increasing prevalence of chronic wounds, resulting in growing challenges in patient care. The high costs of therapy and personnel often limit proper treatment. Chronic wounds significantly impact patients’ quality of life, requiring intensive therapy multiple times per week, along with regular medical checkups. Moreover, they have substantial economic implications, including hospitalizations, personnel costs (eg, wound care nurses, home health care services, physicians, wound managers, day clinics, etc), material costs, and management (eg, medical transportation). Purwins et al [1] identified hospitalizations, nursing staff, and material costs as the main contributors to the overall expenses.

Mobile health (mHealth) apps hold promise for bridging gaps in health care. However, a lack of evidence exists for the effectiveness of available mHealth apps [2], and high-quality trials are needed to examine their effects [3]. So far, only reviews of the use of mHealth apps without systematic searches and evaluations have been performed [4]. To address this gap in evidence for patients with chronic wounds, a systematic analysis is necessary to gather reliable data in this area.

A study by Svendsen et al [5] demonstrated that patient-centered smartphone apps can significantly improve treatment adherence in cohorts of patients with psoriasis and patients with rheumatic diseases. Participants expressed favorable views toward medical apps, indicating a willingness to use such apps if available.

To subjectively assess app quality, the Mobile App Rating Scale (MARS) [6] was developed, which evaluates engagement, functionality, aesthetics, and information. Additionally, the System Usability Scale (SUS) is a 10-item questionnaire for assessing the usability of a system. It has been effectively applied to evaluate mobile apps related to dementia, depression, pediatric obesity, and smoking cessation [7]. Moreover, the Affinity for Technology Interaction (ATI) scale [8] provides an easy and reliable means to quantify an individual’s technology affinity. An aim of this study was to conduct a focused analysis on the core functionalities and core features of the included apps; therefore, we excluded apps with advertisements in order to prevent disruptions in users’ experiences and advertisements’ effects on usability [9]. Advertisements can be confounding variables and make it difficult to compare apps’ performance or users’ experiences. Advertisements can frustrate or annoy users and reduce the clarity and intuitiveness of an app’s interface [10]. We also excluded paid apps to prevent bias toward users with financial means, maintain analysis inclusivity, and ensure that preferences align with intrinsic app quality rather than financial considerations, thereby enhancing the analysis’ fairness and validity.

### Aim of This Study

The primary objectives of this study were to identify and evaluate publicly available smartphone apps designed for patients with chronic wounds. The assessment aimed to provide subjective quality ratings for these apps, while also collecting data on the technical affinity of this specific patient group. To date, a systematic review and assessment of smartphone apps tailored for patients with chronic wounds has not been conducted.

## Methods

### Ethical Considerations

This study was conducted in accordance with the Declaration of Helsinki, and ethical approval was waived by the local ethics committee of the University of Würzburg.

### App Selection

A systematic search of the German Google Play Store and the Apple App Store was performed from April 2022 to May 2022. The search terms used were “wound,” “pressure ulcer,” “ulcus,” “Wunde,” “Wunddokumentation,” and “wound documentation.” Two independent reviewers searched each app store. The inclusion criteria were that apps had to (1) be available in both app stores, (2) be available in English or German, and (3) be specifically designed for patients. Apps that were not available free of charge and apps that contained advertisements were excluded.

The following information, when available in the app stores and on the associated app websites, was collected: app name, target group (eg, patients and medical personnel), cost, platform, advertisements, features, and search term used to identify the app.

### Evaluation of App Quality

The MARS [11] was developed for professional raters to evaluate mobile apps, and it is a validated and reliable scale. The user version of the MARS (uMARS) [12] was designed for users to evaluate the quality of mHealth apps. Both scales are based on a 5-point Likert scale for the following four sections: “Engagement,” “Aesthetics,” “Functionality,” and “Information.” Additionally, there is a “Subjective” section. Studies using the MARS have already been performed for several chronic diseases and apps related to breast cancer. The uMARS [12] has been broadly applied to evaluate apps for rheumatic diseases, weight loss, nutrition tracking, and menstrual tracking. The SUS is a simple, 10-item attitude Likert scale that gives an overall view of subjective assessments of usability [13].

Prior to the evaluation, suitable apps were selected based on the inclusion and exclusion criteria, resulting in a total of 3 apps. The quality of these three apps was then evaluated by 10 physicians using the MARS and SUS. Before the assessment, the physicians first watched a short training video that explained the MARS and were then asked to use the apps for more than 10 minutes.

Finally, the best app—the one with the highest mean MARS score among the physicians—was evaluated by 11 patients with chronic wounds using the uMARS and SUS.

### Evaluation of Technical Affinity

Many studies that evaluate the quality of mobile apps lack information on cohorts’ technical affinity, which is necessary to assess and interpret the results. Therefore, the ATI scale [8] was used to gather information on physicians’ and patients’ technical affinity. The ATI scale was designed to quantify a tendency to actively engage in intensive technology interaction or a tendency to avoid technology interaction. In both the patient group and the physician group, ATI scale scores were collected. A Pearson correlation was used to correlate the ages of patients and ATI scale scores.

### Comparative Analysis of Patients’ and Physicians’ Data

The MARS results represented the physicians’ evaluations, as stated in the *Evaluation of App Quality* section, whereas uMARS results corresponded to the patients’ evaluations. As a next step, after normalization, the Mann-Whitney *U* test was used to analyze whether there was a significant difference between MARS and uMARS scores and between patients’ and physicians’ SUS and ATI scale median scores. A comparison of the five subcategories within the MARS was performed. *P* values of *<*.05 were considered significant. The data analysis was performed with SPSS 23 (IBM Corp).

## Results

### App Selection

**Figure 1.**
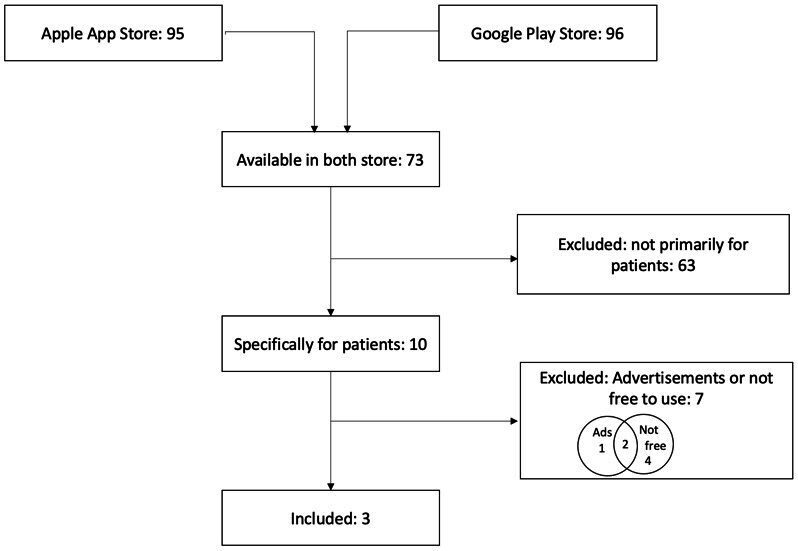
Flowchart illustrating the screening process for identifying suitable mobile apps.

A total of 118 apps were identified—95 in the Apple App Store and 96 in the Google Play Store—of which 73 were available in both app stores (Figure 1). Of these 73 apps, 10 were specifically designed for patients. Of these 10 apps, 1 contained advertising, 4 were not free of charge, and 3 met both exclusion criteria, resulting in a total of 7 apps that were excluded from further analysis.

### Evaluation of App Quality

A total of 3 apps*—WoundEducation*, *APD Skin Monitoring*, and *WUND APP*—met all inclusion and exclusion criteria and were evaluated by 10 physicians. *WoundEducation* provides an overview of different wound types and treatment options. *APD Skin Monitoring* includes an automated area calculation function to quantify wound area. *WUND APP* offers advice and information on chronic wounds and contains a diary function to track patient-related outcomes, such as pain or the level of wound secretion.

The mean MARS scores (and SDs) of the physicians, including subcategory scores and SUS scores, are provided in Table 1.

**Table 1.** Evaluation of *WUND APP*, *APD Skin Monitoring*, and *WoundEducation* by 10 physicians using the Mobile App Rating Scale (MARS) and System Usability Scale (SUS).

Mobile app name	MARS score, mean (SD)	Engagement score, mean (SD)	Functionality score, mean (SD)	Aesthetics score, mean (SD)	Information score, mean (SD)	Psychotherapy score, mean (SD)	SUS score (%), mean (SD)
*WUND APP*	3.88 (0.65)	3.36 (0.89)	4.38 (0.66)	4.13 (0.76)	3.67 (0.56)	2.98 (0.36)	80.5 (17.7)
*WoundEducation*	3.01 (0.5)	2.14 (0.44)	3.93 (0.99)	2.73 (0.75)	3.25 (0.58)	2.57 (0.46)	72.75 (18.5)
*APD Skin Monitoring*	2.64 (0.65)	2.36 (0.56)	2.65 (0.83)	2.77 (0.69)	2.79 (0.82)	2.47 (0.63)	50.75 (27)

*WUND APP* had the highest mean MARS score (mean 3.88, SD 0.65 out of 5) and was subsequently analyzed by 11 patients with chronic wounds. *WUND APP* had a similar mean uMARS score (mean 3.89, SD 0.4 out of 5) when analyzed by the patients.

### Evaluation of Technical Affinity

**Figure 2.**
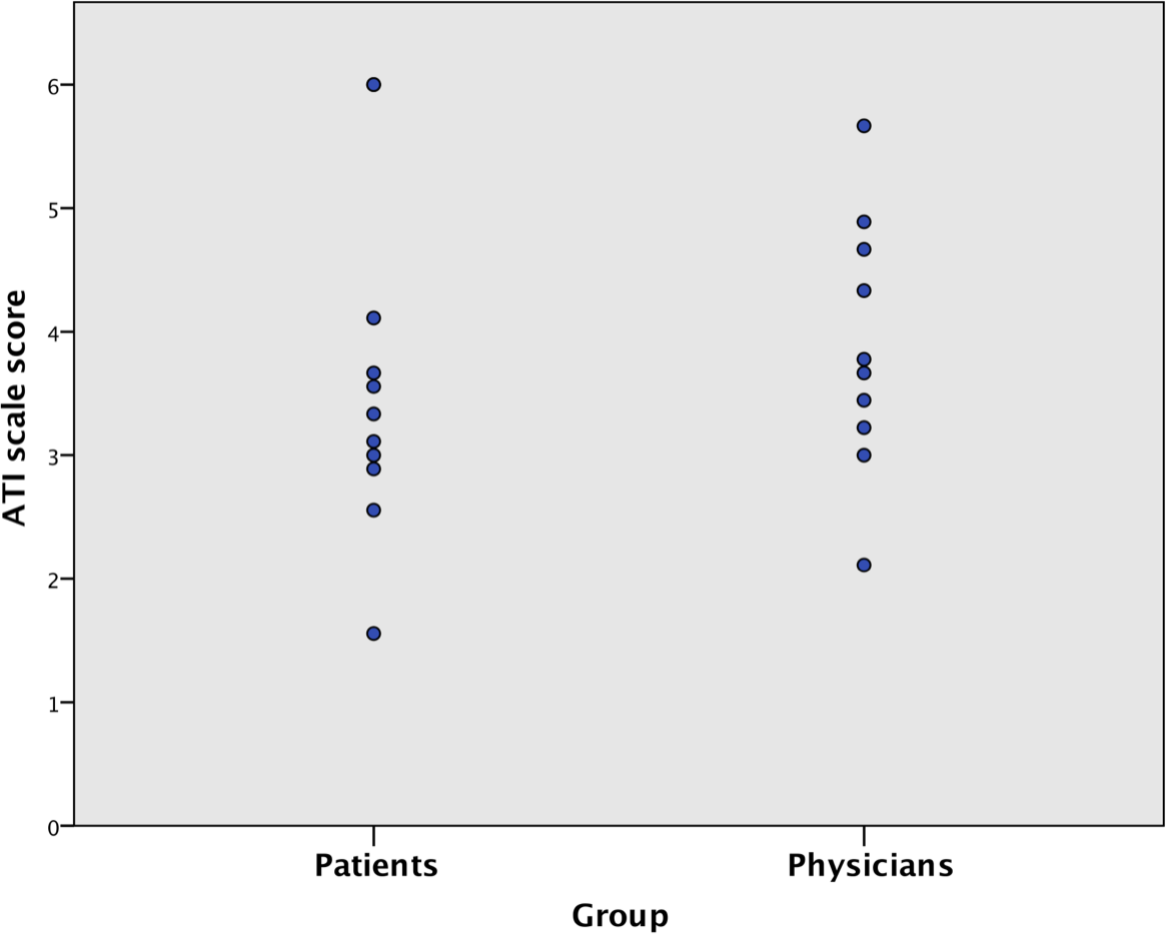
Scatter plot depicting ATI scale scores in patients and physicians. ATI: Affinity for Technology Interaction.

The ATI scale scores ranged from 1.56 to 6 out of 6 for patients and from 2.11 to 5.67 out of 6 for physicians (Figure 2). An ATI scale score of >3 indicates average technology affinity, and a score of >4 indicates high technology affinity [8]. Mean ATI scale scores were slightly lower for patients (mean 3.62, SD 1.35 out of 6) than those for physicians (mean 3.88, SD 1.03 out of 6), but the difference was not statistically significant (*P*=.43). Patients ranged in age from 28 to 70 years; 7 were male, and 4 were female. Physicians ranged in age from 26 to 43 years; 4 were male, and 6 were female. The Pearson correlation coefficient between ATI scale scores and the ages of patients was –0.706 (*P*=.02), indicating a strong negative correlation.

### Comparative Analysis of Patients’ and Physicians’ Data

**Figure 3.**
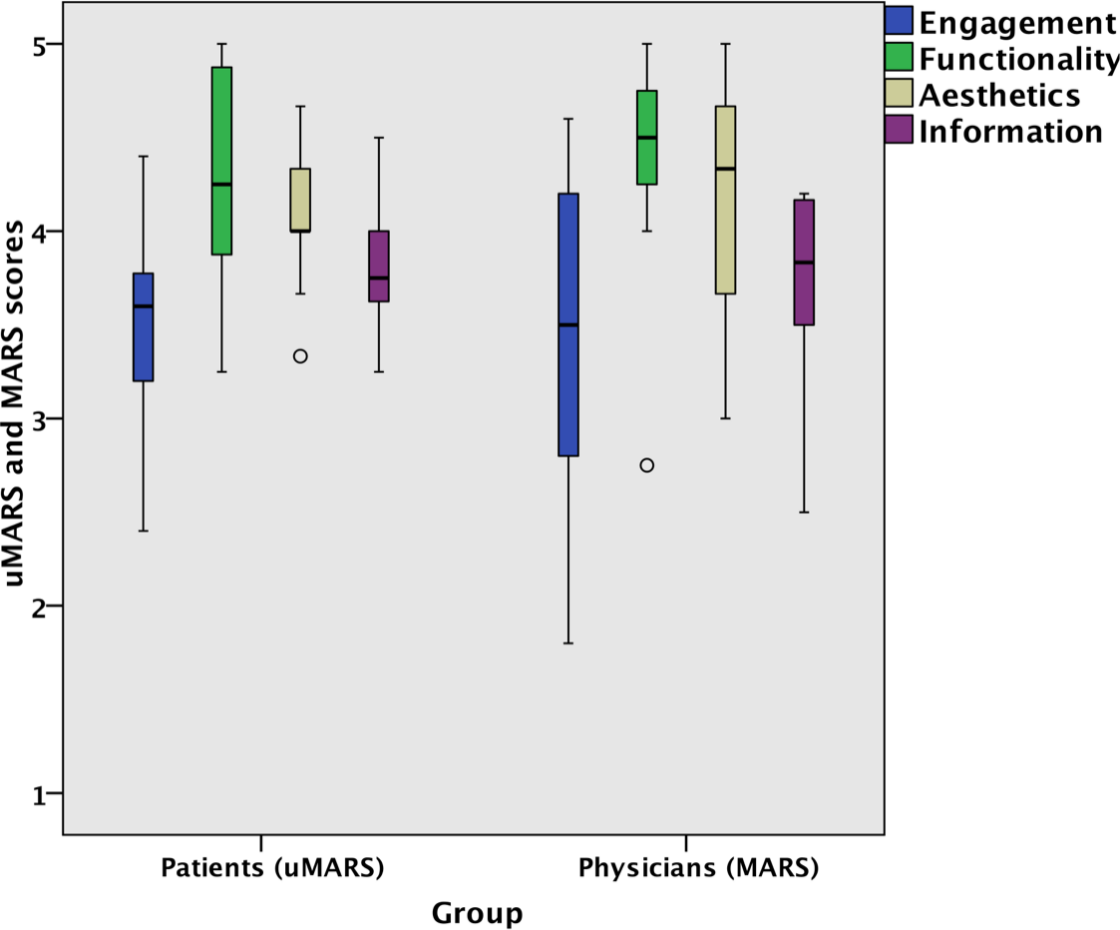
Comparison of MARS and uMARS subscale scores (“Engagement,” “Functionality,” “Aesthetics,” and “Information” scores). Physicians (professional raters) used the MARS, and patients used the uMARS. MARS: Mobile App Rating Scale; uMARS: user version of the Mobile App Rating Scale.

MARS and uMARS scores were almost identical. The subcategory “Information” was rated worse by physicians, while the subcategory “Functionality” was rated worse by patients (Figure 3). For the subcategories “Aesthetics” and “Engagement,” no differences between physicians and patients were found. No statistically significant difference (Mann-Whitney *U* test) was found between MARS and uMARS scores (*P*=.76) or between patients’ and physicians’ SUS scores (*P*=.39; Multimedia Appendix 1).

## Discussion

### Principal Findings

This study represents the first systematic examination that aimed to identify and assess smartphone apps specifically designed for patients with chronic wounds. The quality of these apps was evaluated by independent professional reviewers (physicians) and patients using well-validated scoring systems—the MARS, uMARS, and SUS. Using subjective measures in the assessment of eHealth is relevant and essential, and it is crucial to recognize this fact. Agreement among the assessors, even for subjective measures, is a good sign of the reliability of the assessment [14].

The overall findings indicate that the apps received moderate ratings. Among all apps evaluated, *WUND APP* achieved the highest mean MARS score (mean 3.88, SD 0.65) when assessed by physicians. Similarly, it received a mean uMARS score of 3.89 (SD 0.4) when evaluated by patients. Given that the preselection was exclusively conducted by physicians, patients’ assessments might have differed.

*WUND APP* includes a diary function for documenting wound photos and patient-related outcomes, such as pain or wound secretion. Furthermore, it has a reminder function for necessities such as physician appointments and dressing changes. Its user interface is clear, and the app is user-friendly. Consequently, physicians assigned high scores for the subcategories “Engagement” (mean 3.36, SD 0.89), “Functionality” (mean 4.38, SD 0.66), and “Aesthetics” (mean 4.13, SD 0.76). Additionally, the app provides information on the causes, diagnostics, and treatments of the different wound types, resulting in a mean MARS score of 3.67 (SD 0.56) for the subcategory “Information.”

*WoundEducation* presents information in a straightforward structure that resembles an article with embedded links, delivers medical information, and illustrates various wound types through examples. The app received high ratings for overall functionality (mean 3.93, SD 0.99) and information provision (mean 3.25, SD 0.58). However, it demonstrated limited interactivity, as reflected by having the lowest score for the “Engagement” subcategory (mean 2.14, SD 0.44). Additionally, its aesthetics (mean 2.73, SD 0.75) were rated the lowest among the three apps.

*APD Skin Monitoring* [15] uses a coin as a reference for calculating the area of a wound, proving particularly useful in assessing wound progression and healing status regardless of whether the wounds are irregularly shaped. In addition, the coloration of wounds can be analyzed and tracked over time. However, the app received the lowest ratings for the subcategories “Functionality” (mean 2.65, SD 0.83) and “Engagement” (mean 2.36, SD 0.56).

Our findings revealed that a limited number of wound apps were specifically tailored for patients, accounting for only 14% (10/73) of all wound apps that were available in both app stores. Overall, both physicians and patients rated *WUND APP* similarly; however, although not statistically significant, it is noteworthy that differences emerged in 2 subcategories (“Information”: *P*=.97; “Functionality”: *P*=.56). Physicians rated the information content lower, which could be attributed to their expert medical knowledge. As professionals, they may have had higher expectations regarding the app’s information content and may have found certain aspects less informative. In a recent study, it was shown that patients trusted recommendations and reviews from medical organizations and health care professionals when selecting apps [16]; their motivation to continue using apps was driven by features that supported goal setting and tracking, data sharing, decision-making, and empowerment.

On the other hand, patients rated the functionality of *WUND APP* lower, which was possibly due to their limited involvement in the app’s development process. A systemic review reported that health care professionals were engaged in the development process for only 35% of the 7 analyzed apps that were specifically designed for patients with rheumatoid arthritis [17]. Another systematic review revealed that patients were only engaged in the development process for 15% of the 32 analyzed apps that were designed for individuals with rheumatic and musculoskeletal diseases [18]. The inclusion of patients in the development process of future wound apps could help to ensure that the apps meet their specific needs and preferences. Our study highlights the scarcity of wound apps designed explicitly for patients and the importance of involving patients in the app development process. Tailoring apps to meet patients’ specific requirements and involving them in the design process would likely result in improved app functionality and overall user satisfaction.

To the best of our knowledge, this study represents the first systematic study to collect data on the technical affinity of patients with wounds. Surprisingly, no statistically significant difference in technical affinity was observed between wound care patients and physicians, even with the inclusion of patients aged up to 70 years (*P*=.43). Nevertheless, future studies and app development projects should aim to include older patients and comprehensively assess and address their specific needs. The low adoption and use of mHealth apps among older patients are frequently attributed to inadequate designs [19].

In 2019, Germany introduced a digital health app (DiGA) directory that includes scientifically validated apps. Physicians can prescribe DiGAs in a manner similar to how they prescribe medications [20]. However, to date, no DiGAs specifically tailored for patients with chronic wounds are available in the directory. It is worth noting that physicians with a higher technical affinity and those who are female hold significantly more positive attitudes toward DiGAs [21].

The absence of wound care apps highlights the unmet potential for innovative digital solutions to address the needs of patients with chronic wounds. The inclusion of validated and effective wound care apps in the DiGA directory could significantly improve patient outcomes and health care management in this specific area. It is imperative for future app development initiatives to focus on developing and validating apps that cater to the unique requirements of patients with chronic wounds, to provide them with accessible and effective digital health care resources.

Multiple attempts have been made to integrate mHealth apps into wound care [22], including apps for wound care measurements [23], wound care dressing decision support systems [24], and home-based self-management systems [25]. An Australian study assessed an artificial intelligence app for wound assessment, involving 166 patients in the standard group and 124 in the intervention group. The intervention group demonstrated significantly improved wound documentation, along with positive outcomes such as enhanced patient adherence, efficient digital care provision, and substantial reductions in wound size [26].

A cultural shift toward greater technology affinity has been accelerated by the COVID-19 pandemic [27]. Patients are increasingly using mobile apps when they perceive clear benefits to their use, such as reducing social contact during the COVID-19 pandemic. For patients with chronic wounds, these benefits may include using an app as a diary, while for others, apps may offer the advantage of saving time and transportation costs through telemedicine services. Telemedical approaches could significantly alter and improve the current wound care landscape, bearing the potential to improve the efficiency, accuracy, and accessibility of both diagnoses and treatments. Telemedicine in chronic wound management was shown to be noninferior to conventional standard care in a systematic review and meta-analysis [28]. The earlier diagnosis of complications, such as wound infections, can reduce the use of antibiotics and lower health care costs by preventing hospital stays.

### Limitations

Due to the limited number of apps that met our strict inclusion and exclusion criteria, only a small subset could be analyzed in this study. The selection of apps was conducted within a relatively short time frame, which might have further restricted the available options for evaluation.

The nonsignificant differences between physicians’ and patients’ ratings in this study may be attributed to the small sample of only 11 patients and 10 physicians, which resulted in limited statistical power. Therefore, we emphasize the importance of cautious interpretation and the consideration of larger sample sizes for future research.

Another factor that influenced this study’s results was the inclusion of patients who agreed to evaluate *WUND APP*. This approach potentially introduced selection bias, as our patient sample may not represent the true technical affinity of all patients with chronic wounds. The overall technical affinity of a larger and more diverse patient population might be lower than what was observed in this study. Future studies with larger patient cohorts might provide a more comprehensive understanding of the technical affinity and usability experience of patients with chronic wounds who use mHealth apps. Further, the assessment relied on a qualitative survey—a method that may be susceptible to various biases. However, to truly ascertain the effectiveness of *WUND APP*, a randomized controlled trial is needed.

### Conclusions

Patient involvement is crucial in app development. By involving all stakeholders, including physicians, wound care experts, and patients, throughout the development process, apps can be tailored to meet the specific needs and preferences of the end users, resulting in increased user satisfaction and improved health outcomes.

The validated ATI scale proved to be a valuable tool for evaluating an individual’s technical affinity. In future studies and app evaluations, technical affinity should be determined to generalize outcomes to specific patient and consumer cohorts.

## Supplementary material

10.2196/51592 Multimedia Appendix 1 Patient and physician characteristics (age and sex) and the Affinity for Technology Interaction scale, System Usability Scale, Mobile App Rating Scale (MARS), and user version of the MARS scores (including subcategory scores) of all 11 patients and 10 physicians who participated.

## Abbreviations

ATI: Affinity for Technology Interaction
DiGA: digital health app
MARS: Mobile App Rating Scale
mHealth: mobile health
SUS: System Usability Scale
uMARS: user version of the Mobile App Rating Scale
