# Supplementary material for: Patient-Centered Chronic Wound Care Mobile Apps: Systematic Identification, Analysis, and Assessment
Source: JMIR Mhealth Uhealth. 2024 Mar 21;12:e51592. doi: 10.2196/51592 (PMC11004612; doi:10.2196/51592)
Supplement: Multimedia Appendix 1 [file mhealth-v12-e51592-s001.docx]

| **Patients** | | | | | | **uMARS** | | | | |
| --- | --- | --- | --- | --- | --- | --- | --- | --- | --- | --- |
| **ID** | **Age** | **Sex** | **Platform** | **ATI** | **SUS** | **Engagement** | **Functionality** | **Aesthetics** | **Information** | **Overall Score** |
| 1 | 34 | m | Android | 6.00 | 87.5 | 3.60 | 4.00 | 4.00 | 4.00 | 3.90 |
| 2 | 70 | f | iOS | 1.56 | 55 | 3.75 | 3.75 | 4.33 | 3.50 | 3.83 |
| 3 | 56 | m | iOS | 3.33 | 75 | 3.20 | 4.25 | 4.33 | 3.75 | 3.88 |
| 4 | 57 | m | Android | 3.00 | 70 | 3.60 | 5.00 | 4.33 | 4.00 | 4.23 |
| 5 | 52 | m | Android | 2.56 | 75 | 2.80 | 4.25 | 4.00 | 4.00 | 3.76 |
| 6 | 44 | m | Android | 6.00 | 97.5 | 3.20 | 5.00 | 4.00 | 3.75 | 3.99 |
| 7 | 68 | m | Android | 3.56 | 97.5 | 2.40 | 3.25 | 3.33 | 3.25 | 3.06 |
| 8 | 59 | m | Android | 2.89 | 30 | 3.40 | 3.50 | 3.67 | 3.50 | 3.52 |
| 9 | 58 | f | Android | 3.67 | 50 | 3.80 | 4.00 | 4.00 | 3.75 | 3.89 |
| 10 | 58 | f | Android | 3.11 | 47.5 | 4.40 | 4.75 | 4.67 | 4.50 | 4.58 |
| 11 | 28 | m | Android | 4.11 | 87.5 | 3.80 | 5.00 | 4.67 | 3.75 | 4.30 |
|  | | | | | |  | | | | |
| **Physicians** | | | | | | **MARS** | | | | |
| **ID** | **Age** | **Sex** | **Platform** | **ATI** | **SUS** | **Engagement** | **Functionality** | **Aesthetics** | **Information** | **Overall Score** |
| 1 | 26 | f | iOS | 4.89 | 85 | 3.40 | 5.00 | 4.33 | 4.00 | 4.18 |
| 2 | 44 | f | iOS | 3.00 | 82.5 | 2.20 | 4.25 | 3.00 | 2.50 | 2.99 |
| 3 | 31 | m | iOS | 5.67 | 75 | 3.60 | 4.50 | 4.33 | 3.50 | 3.98 |
| 4 | 32 | f | Android | 3.44 | 72.5 | 2.80 | 4.25 | 4.67 | 3.67 | 3.85 |
| 5 | 30 | f | iOS | 4.33 | 92.5 | 4.20 | 4.75 | 4.67 | 4.17 | 4.45 |
| 6 | 30 | f | Android | 3.22 | 95 | 4.60 | 4.75 | 5.00 | 4.17 | 4.63 |
| 7 | 30 | m | Android | 4.67 | 85 | 3.20 | 4.00 | 3.67 | 3.50 | 3.59 |
| 8 | 36 | f | Android | 3.78 | 35 | 1.80 | 2.75 | 3.00 | 3.00 | 2.64 |
| 9 | 29 | f | iOS | 3.67 | 95 | 4.20 | 4.50 | 5.00 | 4.00 | 4.43 |
| 10 | 29 | m | iOS | 2.11 | 87.5 | 3.60 | 5.00 | 3.67 | 4.20 | 4.12 |
